# Hodgkin’s lymphoma presenting as a complex paraneoplastic neurological syndrome: a case report

**DOI:** 10.1186/1752-1947-7-96

**Published:** 2013-04-08

**Authors:** Nischala Ammannagari, Shailaja Chikoti, Eric Bravin

**Affiliations:** 1 Department of Internal Medicine, Bassett Medical Center, Cooperstown, NY 13326, USA; 2 Division of Hematology and Oncology, Bassett Medical Center, Cooperstown, NY, 13326, USA

## Abstract

**Introduction:**

Paraneoplastic neuropathies are rare. They are often difficult to diagnose, especially when they precede the diagnosis of cancer. Hodgkin's lymphoma is associated with multiple paraneoplastic neurological syndromes, of which demyelinating polyneuropathies are very unusual. Association with chronic inflammatory demyelinating polyneuropathy is even more uncommon.

**Case presentation:**

We report the rare case of a 74-year-old Caucasian man who presented with a complex neurological syndrome and was eventually diagnosed with the nodular sclerosing variant of Hodgkin's lymphoma. With timely diagnosis and early institution of treatment of the underlying malignancy, our patient began to show gradual improvement of his symptoms.

**Conclusion:**

Hodgkin's lymphoma is associated with several paraneoplastic neurological syndromes. Sometimes it can be the only presenting feature of an underlying Hodgkin's lymphoma, posing a diagnostic challenge. Prompt oncologic treatment and immunotherapy can be beneficial if instituted early in the course of the disease.

## Introduction

Hodgkin’s lymphoma is associated with multiple paraneoplastic neurological syndromes. Association with chronic inflammatory demyelinating polyneuropathy (CIDP) is very rare. Sometimes it can be the only presenting feature of an underlying Hodgkin’s lymphoma, posing a diagnostic challenge. We present one such rare case of a 74-year-old Caucasian man who presented with a complex neurological syndrome and was eventually diagnosed with nodular sclerosing Hodgkin’s lymphoma. With timely diagnosis and early institution of treatment of the underlying malignancy, our patient began to show gradual improvement of his symptoms.

## Case presentation

A 74-year-old Caucasian man with past history of chronic lymphocytic leukemia (CLL) currently in remission presented to our primary care clinic with drooping eyelids, double vision, generalized weakness, proximal muscle weakness and imbalance of two weeks’ duration.

A physical examination revealed ptosis, horizontal nystagmus on right gaze and ataxia. To evaluate for possible brain stem stroke, a computed tomography scan of his head without contrast and magnetic resonance imaging (MRI) of his brain were done and found to be normal. He was then referred to a neurologist. A lumbar puncture was performed and cerebrospinal fluid analysis revealed a white count of 7, glucose level of 91mg/dL, protein level of 74mg/dL, no malignant cells and a negative Lyme titer. The Miller-Fisher variant of Guillain-Barré syndrome was suspected. However, anti-ganglioside antibody GQ1b was found to be negative. With a strong suspicion for an inflammatory demyelinating polyneuropathy, our patient was empirically started on systemic steroids. He received a full two-week course of oral prednisone 60mg daily and was then tapered off the steroids slowly. He showed significant clinical improvement except for mild persistent generalized weakness.

Three months later, our patient again presented to his neurologist with worsening of the ptosis and generalized weakness. He denied any dysphagia. Myasthenia gravis was suspected. However, acetylcholinesterase antibodies were found to be negative. A nerve conduction study was scheduled.

Meanwhile, within the next two days, he developed worsening weakness of all extremities, associated with severe worsening ptosis, diplopia and paresthesias. He was admitted to our inpatient medicine service. He denied any dysphagia, dyspnea or slurred speech. He did not recall any recent infectious illness.

Our patient’s past history was significant for a diagnosis of CLL 10 years previously during an evaluation of a asymptomatic elevation of his white blood cell count. He was treated with four cycles of fludarabine and cyclophosphamide four years previously for symptomatic thrombocytopenia and had been in remission since then.

On admission to our inpatient medicine service, a physical examination revealed ptosis, vertical and horizontal nystagmus, diminished strength in all extremities, hyporeflexia, and impaired sensation to touch and vibration. Bilateral Babinski signs were absent. His coordination was normal and his gait was stable with a walker.

Laboratory tests including a complete blood count, complete metabolic panel, thyroid function, vitamin B12, folate, glycosylated hemoglobin, aldolase, creatine phosphokinase and a urinalysis were unremarkable. A repeat MRI scan of his brain was normal.

**Figure 1:**
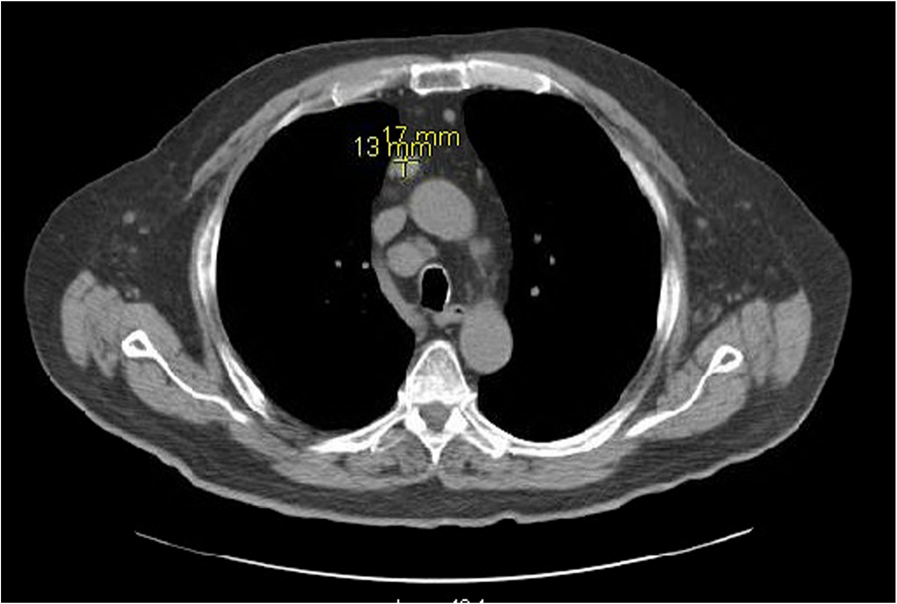
Computed tomography of the thorax showing the anterior superior mediastinal node measuring 1.7×1.3cm.

**Figure 2:**
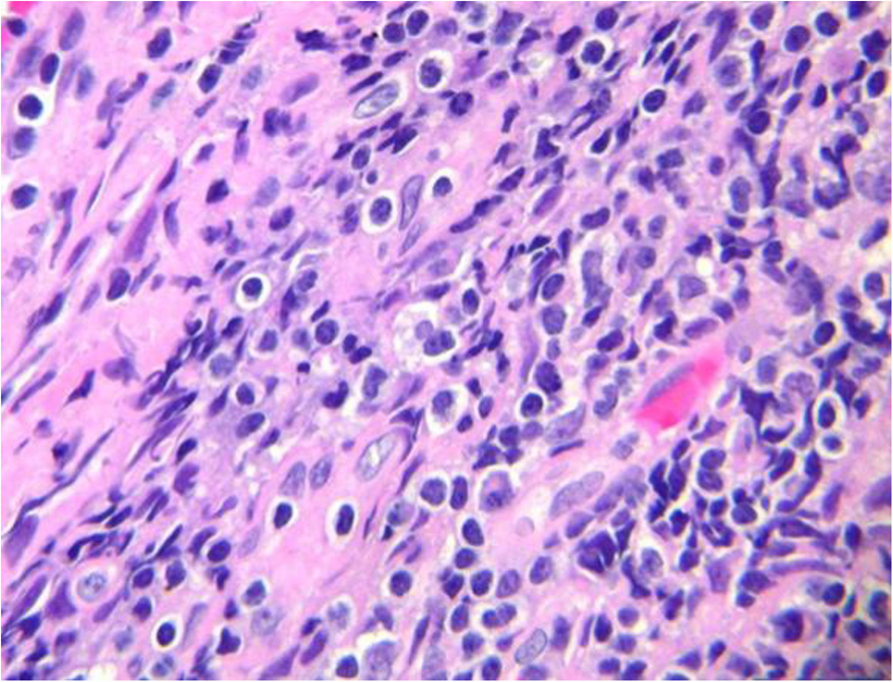
Nodular sclerosing pattern of Hodgkin’s lymphoma.

An electromyogram and nerve conduction studies revealed slow nerve conduction and moderately severe demyelinating motor and sensory polyneuropathy involving bilateral peroneal, sural, median and ulnar nerves. MRI of the spine revealed degenerative changes of the lumbar spine but no evidence of any abnormal enhancement of nerve roots. A computed tomography scan of chest and thorax to evaluate for any mediastinal pathology revealed mediastinal lymphadenopathy measuring 1.7×1.3cm (Figure 1). A mediastinoscopy and lymph node biopsy revealed nodular sclerosing Hodgkin’s lymphoma (Figure 2). A paraneoplastic panel for neuropathies including anti-Hu, Yo, Tr, Ma1, Ma2, Ri, cancer-associated retinopathy, Lambert–Eaton myasthenic syndrome (LEMS), CV2, Zic4, voltage-gated potassium channels, amphiphysin and G-acetylcholine receptor antibodies were all negative (Table 1).

**Table 1:** Well-established paraneoplastic antibodies with the associated paraneoplastic syndromes and associated cancers

**Antibody**	**Paraneoplastic neurological syndrome**	**Associated cancers**
Hu	Sensory neuropathy, encephalomyelitis	Neuroblastoma, SCLC
Yo	Cerebellar degeneration	Breast, ovarian
Tr	Cerebellar degeneration	Hodgkin’s lymphoma, thymoma
Ma	Cerebellar, hypothalamic, limbic encephalitis	Testicular, breast, multiple other cancers
Ri	Cerebellar degeneration, opsoclonus, brainstem encephalitis	Breast, gynecological, SCLC, Hodgkin’s, neuroblastoma
Cancer-associated retinopathy	Photoreceptor degeneration	SCLC
Lambert-Eaton myasthenic syndrome	Lambert-Eaton myasthenic syndrome	SCLC
CV2	Cerebellar degeneration, encephalomyelitis, peripheral neuropathy	Germ cell tumors of testis, SCLC
Zic4	Cerebellar degeneration	SCLC
Voltage-gated potassium channels	Seizures, limbic encephalitis	Thymoma
Amphiphysin	Stiff person syndrome, encephalomyelitis	Breast, lung cancer
Acetylcholine receptor antibodies	Myasthenia gravis	Thymoma
Recoverin	Retinopathy	SCLC

SCLC; small cell lung cancer.

Initially upon hospitalization, our patient was treated with intravenous immunoglobulin for suspected myasthenia gravis with no clinical improvement. However, after the diagnosis of Hodgkin’s lymphoma was made, he was started on chemotherapy with adriamycin, bleomycin, vinblastine and dacarbazine. At the time of writing, our patient has completed six cycles of this chemotherapy regimen and his neurological symptoms are improving gradually.

## Discussion

Paraneoplastic neuropathies are seen in association with 4% to 5% of cancers [1-3]. Hodgkin’s lymphoma is also associated with multiple paraneoplastic neuropathies like cerebellar degeneration, acute inflammatory demyelinating polyneuropathy (Guillain-Barré), CIDP, chorea and ataxia, subacute sensory neuropathy, motor neuron disease, myasthenia gravis, stiff person syndrome and brachial neuropathy [1,4]. The most common neurological syndrome described in the literature is subacute cortical cerebellar degeneration with more than 50 cases reported so far [1,5,6]. Demyelinating neuropathies are very rare [3,7-9]. Few cases of CLL associated with paraneoplastic demyelinating neuropathies like Guillain-Barré syndrome or Miller-Fisher syndrome are reported to date [10-13].

In our patient, the prolonged clinical course, significant sensory impairment and slow nerve conduction in addition to negative GQ1b antibody favor the diagnosis of CIDP over acute inflammatory demyelinating polyneuropathy [2,7,14].

According to the European Federation of Neurological Societies and the Peripheral Nerve Society guidelines, diagnosis of CIDP requires a typical or atypical clinical picture with clear demyelinating electrodiagnostic changes in two nerves, or probable demyelinating features in two nerves plus at least one supportive feature (from cerebrospinal fluid analysis, nerve biopsy, MRI or treatment response to immunotherapy). In our patient, an electromyogram showed demyelinating changes involving more than two nerves, fulfilling the diagnostic criteria [15].

Neurolymphomatosis was thought to be unlikely in this case given the normal cerebrospinal fluid findings and negative MRI findings. Also, neurolymphomatosis is commonly reported in association with non-Hodgkin’s lymphoma and not Hodgkin’s lymphoma [16].

CLL only rarely transforms into Hodgkin’s lymphoma as seen in our patient [17-20]. In this case, we hypothesize that our patient’s CLL could have transformed into nodular sclerosing Hodgkin’s lymphoma, which presented with paraneoplastic neuropathy as its initial manifestation [17].

The pathogenesis of paraneoplastic demyelinating neuropathies is not clear although autoimmune mechanisms have been implicated. Paraneoplastic syndromes have been linked with the production of antibodies stimulated by tumor antigens that consequently target the neuronal antigens. Examples include anti-Hu associated with small cell lung cancer (SCLC) and neuroblastoma, anti-Yo with breast and ovarian cancer, anti-Tr with Hodgkin’s lymphoma, anti-Ma with testicular cancer, anti-Ri with breast cancer and neuroblastoma, anti-amphiphysin with breast cancer, Lambert-Eaton myasthenic syndrome antibody with SCLC, recoverin with SCLC and so on [7] (Table 1, [21,22]). However, these antibodies might not be positive in all cases of paraneoplastic neurological syndromes as seen in our patient [23].

Because the majority of neurologic paraneoplastic syndromes are immune-mediated, two general approaches to therapy are recommended: removal of the antigen source by treatment of the underlying malignancy and suppression of the immune response. There is evidence that prompt oncologic treatment and immunotherapy can be beneficial, especially if instituted during the time of symptom progression rather than after deficits have been fully established [24,25].

## Conclusion

Hodgkin's lymphoma is associated with multiple paraneoplastic neurological syndromes, of which CIDP is very uncommon. Sometimes, this can be the only presenting symptom of an underlying Hodgkin’s lymphoma, posing a diagnostic dilemma. However, when diagnosed on time, it may afford an opportunity for the institution of early treatment of the underlying malignancy, with favorable outcome.

## Consent

Written informed consent was obtained from the patient for publication of this case report and accompanying images. A copy of the written consent is available for review by the Editor-in-Chief of this journal.

## Abbreviations

CIDP: Chronic inflammatory demyelinating polyneuropathy; CLL: Chronic lymphocytic leukemia; MRI: Magnetic resonance imaging; SCLC: Small cell lung cancer.

## Competing interests

All the authors declare that they have no competing interests.

## Authors’ contributions

All authors had access to the data and contributed to the content of the case report. NA and SC were involved in the patient care during his admission to the hospital that led to subsequent diagnosis. EB was the primary oncologist for the patient. NA and SC wrote the manuscript and EB mentored and supervised the entire case writing. NA was also responsible for the entire literature search. All authors read and approved the final manuscript.
